# Canine Crown Sexual Dimorphism in a Sample of the Modern Croatian Population

**DOI:** 10.3390/dj11070175

**Published:** 2023-07-18

**Authors:** Jelena Dumančić, G. Richard Scott, Ivana Savić Pavičin, Sandra Anić-Milošević, Nataša Medančić, Hrvoje Brkić

**Affiliations:** 1 Department of Dental Anthropology, School of Dental Medicine, University of Zagreb, 10000 Zagreb, Croatia; savic@sfzg.hr (I.S.P.); brkic@sfzg.hr (H.B.); 2 Department of Anthropology, University of Nevada, Reno, NV 89557, USA; grscott@unr.edu; 3 Department of Orthodontics, School of Dental Medicine, University of Zagreb, 10000 Zagreb, Croatia; sanic@sfzg.hr; 4 Private Dental Practice, 10000 Zagreb, Croatia; natasamedancic@hotmail.com

**Keywords:** sex identification, forensic dentistry, forensic anthropology, dental anthropology, canine

## Abstract

Sex assessment is a key part of forensic analysis to establish the identity of unknown deceased individuals. Previous studies have shown that canines are the most dimorphic teeth, but population-specific data are necessary for forensic methods. This study explores sex dimorphism in canine crown dimensions and morphology in a contemporary Croatian population. The material consisted of 302 dental casts (147 females, 155 males) of orthodontic patients and dental students (11–25 years). The distal accessory ridge (DAR) of the upper and lower canines was evaluated using the Arizona State University Dental Anthropology System. Mesiodistal (MD) and buccolingual (BL) crown dimensions were measured on 120 casts. Sex differences in MD and BL dimensions were significant (*p* < 0.05) for all the canines (upper and lower, left and right), while in DAR only for lower canines (*p* < 0.000001). When all variables were put into the model, backward stepwise discriminant function analysis isolated lower canine DAR and lower left canine MD as the two independent variables differentiating sex. Using these two variables, a discriminant function formula allowed for sex determination with an accuracy of 73.5%. This study shows that both canine crown morphology and dimensions are useful for sex determination, especially for lower canines. These methods can be applied to children, as lower canines erupt at about 9 years of age.

## 1. Introduction

Anthropological analysis for establishing the biological profile of unknown deceased individuals includes sex, age, and height estimation. In forensic cases, sex determination is critically important, as by identifying sex, we are eliminating 50% of the population from the identification process. Furthermore, after sex estimation, it is possible to conduct sex-specific age estimation [1]. For sex identification, osteological analysis most commonly uses morphological characteristics of the pelvis and skull [2]. The analysis of mandibles also proved successful in sex determination [3,4]. This can be hindered by bone fragmentation, poor preservation, and the complexity of the human skeleton [3,5]. Furthermore, as skeletal sexual characteristics develop during puberty, sex estimation from bone characteristics is not possible before the age of 16–18 years [6]. DNA analysis is the most reliable method for sex identification, but it is a prolonged and expensive procedure that also results in the destruction of a part of the sample [5].

Dentition can serve as a valuable source of information on the human biological profile, as teeth are readily available and data collection is simple [1]. Furthermore, teeth are durable in the postmortem environment and usually remain the best-preserved part of the human skeleton [7]. Sometimes, they are the only source of information that can be used (burnt remains, poorly preserved remains, and fragmented remains). The development of permanent teeth takes place early in childhood, and the influence of sex chromosomes is expressed in the sexual dimorphism of tooth crowns and roots before puberty and the development of skeletal sexual characteristics [8]. Dental anthropological methods of sex assessment include the analysis of tooth size and morphology.

Differences in tooth size are assessed by odontometrics, traditionally comprised of linear measurements of tooth dimensions. Newer technologies include the application of 3D optical scanners, micro-CT, and Cone Beam Computed Tomography (CBCT) with the provision of automated measurements on 3D and 2D images [9,10]. Previous studies in modern human populations have shown that male teeth are 2–6% larger than female teeth [11]. This difference is small, but with the application of statistical methods, usually discriminant function analysis, it is possible to correctly classify sexes in 77% to 87% of the cases [11,12]. Two studies report precision of nearly 95% [13,14]. Logistic regression analysis proved to be more successful than discriminant function analysis even in incomplete dentitions, while enabling optimal sex prediction (100%) when all teeth in both jaws were included [15].

The other approach to sex estimation by teeth is to analyze morphology. The term tooth morphology refers to traits that are present or absent and, when present, exhibit variable degrees of expression [16]. For the analysis of non-metrical variation of dental traits, it is necessary to use ranked standards for scoring, and the most widely used is the Arizona State University Dental Anthropology System (ASUDAS) [17,18]. Sexual dimorphism in tooth morphology, crown, and root traits has been tested many times, and the results vary from author to author. If a significant sex difference is found in the expression of a characteristic, then it is usually more often and more strongly expressed in males. It is possible that the size of the teeth has an influence here [16]. The only trait that shows consistent sex dimorphism across different samples is the distal accessory ridge (DAR) of the upper and lower canines [19,20].

As human tooth crowns and roots show geographic variation both in size and morphology, population-specific data are required for forensic applications. The present study explores sex dimorphism in the canine DAR and crown dimensions in a contemporary Croatian population to develop a formula for sex determination. The hypothesis assumes that both canine crown morphology and dimensions can be used for sex estimation.

## 2. Materials and Methods

The material consisted of 302 dental casts of dental students and orthodontic patients from the collections of the Departments of Dental Anthropology and Orthodontics, School of Dental Medicine, University of Zagreb, Croatia. There were 147 females and 155 males, with an age range of 11–25 years. Dental exams and impressions were taken between 2014 and 2021. Informed consent was obtained from all participants and legal guardians. The research is a part of the larger research project “Analysis of teeth in forensic and archaeological research”, approved by the Ethics Committee of the School of Dental Medicine, University of Zagreb.

**Figure 1:**
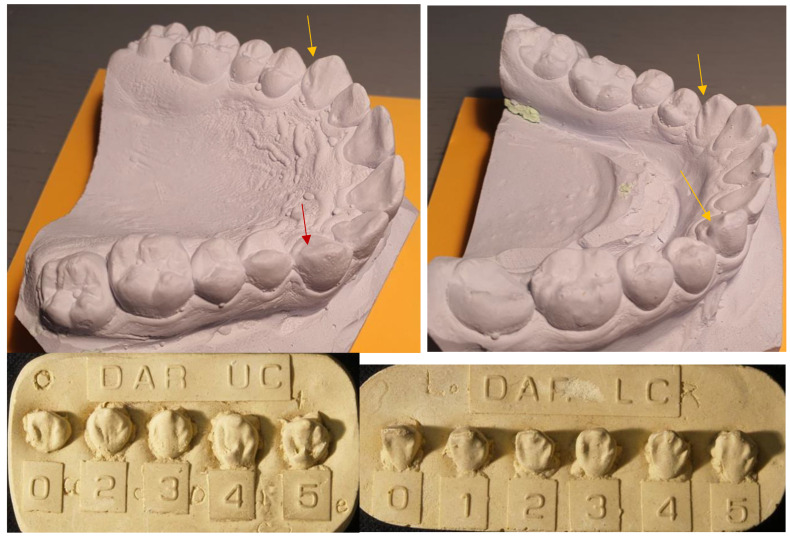
Case 29 with the ASUDAS referent plaques for scoring the distal accessory ridge (DAR) of the upper (plaque DAR UC) and lower canines (plaque DAR LC). In this case, the upper right canine and both lower canines were scored as grade 4 (yellow arrows). The upper left canine was not scored due to wear (red arrow).

The morphological variant, the Distal Accessory Ridge (DAR), of the upper and lower canines was scored on the total sample following the guidelines of the Arizona State University Dental Anthropology System (ASUDAS). DAR is defined as the accessory ridge that appears in the distolingual fossa between the tip of the cusp and the distolingual marginal ridge [18]. Figure 1 presents case 29 with the ASUDAS reference plaques for scoring DAR of the upper and lower canines. Assessment was performed by a single experienced examiner (J.D.) who was blinded to the sex of the cases. Although all canines were assessed, for statistical calculations the individual count method was used, where the antimere with the highest degree of expression is scored per individual case [17]. This method maximizes sample size, avoids the problem of antimeric symmetry, and holds that the side exhibiting the greatest trait expression best reflects the underlying genotype. Canines manifesting wear facets in the area of the DAR were excluded from scoring and recorded as missing data.

Odontometric measurements reported here were taken from a previously conducted study on 120 casts from the orthodontic patient’s sample, which was a part of the same research project [21]. Mesiodistal (MD) and buccolingual (BL) crown dimensions were measured by a single examiner (N.M.) using a digital caliper with an accuracy of 0.1 mm.

The FDI tooth notation system was used.

### 2.1. Statistical Analysis

Statistical analyses were performed using STATISTICA data analysis software system version 12 (StatSoft, Inc., 2013, Tulsa, OK, USA) and MedCalc^®^ Statistical Software version 20.115 (MedCalc Software Ltd., 2022, Ostend, Belgium). Nominal variables were presented as numbers and ratios (%), and continuous variables were presented as median and interquartile range (IQR). The differences according to sex and rank of the canine DAR were tested using Kruskal-Wallis ANOVA for ranks. Sex differences in the canine dimensions were tested using the Student’s *t*-test. The correlation between upper and lower canine DAR was tested using Spearman’s correlation. The variables differentiating sex were tested using discriminant function analysis using the backward stepwise approach. All analyses were considered statistically significant with a *p* < 0.05.

### 2.2. Intraobserver Error Calculation

For the calculation of intraobserver error rates in scoring the distal accessory ridge, observations were repeated on 31 casts at a three-month interval. The error was calculated as proposed by Nichol and Turner [22]. Five values were calculated: (1) disagreement as to whether certain casts could be observed for the trait (Observed Only one Session %); (2) the percentage of casts scored in both sessions with disagreements (Variant Scoring); (3) the percentage of disagreements that are of two grades or more (>1 Grade Variant Scoring); (4) the Absolute Mean Grade Difference (AMGD) representing the average difference, expressed in percentage of a grade made for the individual cast scored, ignoring the direction of the difference (see formula below); and (5) the Net Mean Grade Difference (NMGD) representing the average difference, expressed in percentage of a grade, made for the individual cast scored, which takes into account the directionality of scoring discrepancies (see formula below). If the value is near zero, then discrepancies are random, or nearly so.

$$AMGD=\frac{\sum (|X2-X1|)}{n}*100$$

$$NMGD=\frac{\sum (X2-X1)}{n}*100$$

The calculated errors were: (1) Observed only one session = 8% of casts; (2) Variant scoring = 25% of casts; (3) >1 Grade variant scoring = 0 casts; (4) AMGD = 25% of a grade; and (5) NMGD = 4% of a grade.

## 3. Results

### 3.1. Sex Dimporphism in Canine Distal Accessory Ridge

**Figure 2:**
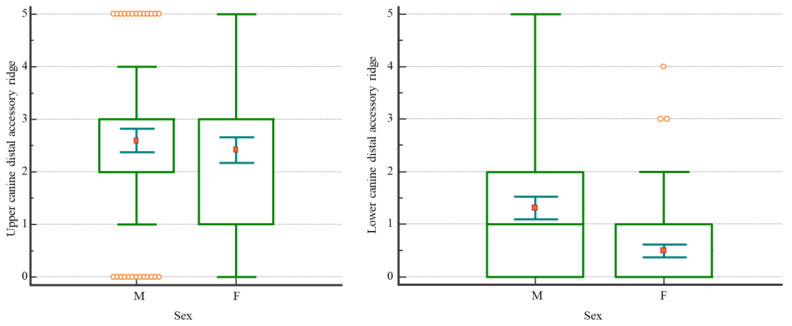
Box whisker plots showing the upper and lower canine distal accessory ridges by sex and grade of expression (mean values, minimum, maximum, 25th, and 75th percentiles). Orange circles represent outlier cases.

Descriptive statistics for the upper and lower canine DAR are presented in Table 1. The distribution of data by DAR grade of expression and by sex is presented in Figure 2. Sex dimorphism was tested by Kruskal-Wallis ANOVA for ranks, which found a significant difference only for lower canines (Table 2).

The correlation of the upper and lower canine DAR proved to be significant (number of individuals with scored upper and lower canines = 287, Spearman’s correlation coefficient = 0.413, significance level *p* < 0.0001).

### 3.2. Sex Dimorphism in Odontometric Measurements

Table 3 shows odontometric data. The Student’s *t*-test revealed significant sex differences in MD and BL dimensions for all the canines (upper and lower, left and right).

### 3.3. Discriminant Function Analysis of the Variables Differentiating Sex

The variables differentiating sex, all upper and lower canine dimensions, and lower canine DAR, were tested using Discriminant Function Analysis using the backward stepwise approach. Two variables turned out to be independently and statistically significantly correlated to sex: lower canine DAR and lower left canine mesiodistal crown width (Table 4).

The Discriminant Function Analysis provided formulas for both sexes.

Male = −122.854 + 0.435 × * lower canine DAR + 35.568 × 33MD

Female = −111.107 + (−0.326 × lower canine DAR) + 33.908 × 33MD

For each case, variable values are to be put in both formulas. The higher score indicates the predicted sex: if Male formula result > Female formula result, the case is predicted to be male. It was possible to correctly identify 64.4% of males and 82.8% of females, with an overall accuracy rate of 73.5%.

## 4. Discussion

The aim of the present study was to assess sexual dimorphism in permanent canines and to produce a formula for sex determination for the Croatian population. To the authors’ knowledge, this is the first study on canine sexual dimorphism in a Croatian population with such a large sample. Dental casts from individuals younger than 26 years with fully erupted canines were chosen to avoid the influence of tooth wear, which can obscure morphological details and the degree of trait expression. Another advantage of this study is that we assessed sexual differences in both canine size and morphology. For the assessment of morphology, ASUDAS was used as the widely recognized standard, which allows comparison with other populations and inclusion in the database of worldwide variations in dental morphology. The recommended individual count methodology was used, which maximizes sample size, avoids the problem of antimeric symmetry, and holds that the side exhibiting the greatest trait expression best reflects the underlying genotype. As to intraexminer scoring error, there was a 25% Variant Scoring difference, which is expected when there is only one grade difference, as if the trait is between two grades of expression, it can be scored with a lower or upper grade each time randomly. There were no scores with more than one grade difference, which would be an error of concern. The average Mean Grade Difference between scorings was small, 25% of a grade, and the Net Mean Grade Difference was only 4% of a grade, showing there was no direction in scoring differences. According to Nichol and Turner, all values showed low intraobserver error [22].

Kruskal-Wallis ANOVA for ranks revealed significant differences between sexes in DAR, but only for the lower canine (Table 2), which showed higher expression in males (*p* < 0.000001, Table 1, Figure 2). Upper and lower canine DAR were significantly correlated (*p* < 0.0001, Spearman’s correlation coefficient). Odontometric measurements revealed that all canine dimensions were significantly smaller in females (Table 3). The difference varied from a minimal 3.05% to a maximal 5.23%. Finally, discriminant function analysis allowed for sorting out two variables that turned out to be independently and statistically significantly correlated to sex: lower canine DAR and lower left canine mesiodistal crown width (Table 4). With the application of these variables, it was possible to correctly identify sex in 73.5% of the cases. Our research hypothesis was confirmed.

One can question why sex determination was more successful in females (82.8%) than in males (64.4%), while DAR was more pronounced in males. The distribution of the lower canine DAR by grade of expression and sex (right box whisker plot in Figure 2) shows that there is a large overlap of distributions of grades 0 and 1 in both sexes: more than 50% of males and more than 75% of females express grades 0 and 1. However, the distribution in females is more compact, with only three outlier cases above grade 2. The distribution in males is more widely spread, with about 20% of cases above grade 2, and this might explain why sex determination was less successful in males. This does not impact our results. The nature of discriminant analysis is that it draws the “line” between two or more groups. The crucial criteria for successful analysis are overall accuracy and the significance of discriminant function. It is irrelevant which group is classified better.

The limitation of our study is that derived formulas have yet to be tested. It is possible that fewer correct classifications would be achieved on a new sample. Moreover, in people older than 25 years and even in younger individuals, dental wear can erase DAR and reduce the applicability of canine morphology. Interestingly, recent research by Luna showed successful application of the formula for sex assessment derived from canine dimensions in a Portuguese sample from the late 19th and early 20th centuries on an archaeological sample from the Roman period [7].

In newly published research, from which we took odontometric measurements, an artificial neural network model was prepared that used 12 odontometric variables of the upper and lower canines (MD, BL, and cervicoincisal measurements) for predicting the sex [21]. The accuracy achieved was 72.0–78.1%. This is similar to our result using only lower canine morphology and MD dimensions. Not much is added by including upper canines, cervicoincisal measurements, and the artificial intelligence algorithm. However, after adding the orthodontic variables anterior Bolton ratio and age, the percentage of accurate predictions increased to 77.8–85.7%.

Our previous research on the same topic was conducted on a different sample of 160 dental casts from the old archive of the Department of Dental Anthropology collected during the 1970s and 1980s [23]. Log-linear analysis showed significant sex differences in the distal accessory ridge for both upper (*p* < 0.05) and lower canines (*p* < 0.000005). While results are the same regarding sex dimorphism of the lower canines, the difference for the upper canine can be explained by possible differences between the samples, differences in statistical methods, a possible error in conclusion based on a low *p*-value (*p* < 0.05), or a larger intraobserver error of 7.7% scoring differences of two grades or more. We consider the latter two reasons the most probable due to the relatively smaller sample and larger intraobserver error.

Although our discriminant function analysis final model did not include maxillary canines, as lower canines were better discriminators, other authors’ methods resulted in successful determination of sex using only upper canines. García-Campos et al. studied volumes and surface areas of enamel and dentin in maxillary canines from 56 individuals of different geographic origins using micro-CT [10]. They found thicker enamel in females and thicker dentine in males, leading to a difference in dental size in favor of males. Discriminant functions allowed for successful identification of sex in between 87.5% and 93.75% of the cases. This is a better result than the same statistical method yielded in our research. It is probably due to the higher precision of micro-CT measurement compared to odontometry on dental casts. However, micro-CT requires expensive equipment that is not available to all and is not suitable for research on living individuals due to radiation. Dental casts, on the other hand, are valuable records of the contemporary population and are readily available at dental schools due to clinical work or can be taken with minimal discomfort and cost.

Noss et al. investigated the influence of tooth size on dimorphism in the Carabelli trait and the canine DAR in Pima Indians [24]. Although size dimorphism contributed to morphologic dimorphism, they concluded that other factors unrelated to crown size contributed the majority of the variance in trait expression. This finding is concordant with our research, where discriminant analysis sorted out morphology (lower canine DAR) and size (mesiodistal dimension of the lower left canine) as independently and statistically significantly correlated to sex, so both contribute to sex determination.

Viciano et al. conducted odontometric sex estimation on three populations of the Iron Age from Italy [25]. They developed logistic regression formulas based on permanent tooth measurements of adult individuals whose sex had been estimated based on pelvic and cranial features. The mandibular canine showed the greatest sexual dimorphism, followed by both maxillary and mandibular first and second molars. The formulas were applied to the permanent dentition of children and adults whose sex could not be estimated with anthropological methods, with an applicability rate of about 80%. This is concordant with our findings, where lower canines showed greater sexual dimorphism than upper canines.

Vodanović et al. investigated sex dimorphism in tooth dimensions in a medieval sample from Croatia and found the greatest significant differences for the upper canine buccolingual diameter of the crown and mesiodistal diameter of the tooth neck [26]. For the lower canine, there were no significant differences, which is contrary to our results. Although it is assumed that the archaeological sample was of a Slavic population, and contemporary Croats are Slavic people, centuries that divide populations may result in the differences. Furthermore, small sample sizes in the archaeological research may have contributed to the different results.

Abrantes et al. studied sex differences in dental morphological characteristics with the ASUDAS methodology on dental casts of 110 orthodontic patients from a Portuguese population [27]. The lower right canine proved to be the most dimorphic tooth. A sex classification model using significantly different morphology traits of teeth 13, 27, and 43 was successful in sex classification in 76.4% of the cases. The methodology in this research (ASUDAS applied to dental casts of contemporary individuals) is comparable to ours, and success in sex determination is very similar to the overall accuracy rate of 73.5% achieved in our study.

Angadi et al. measured mesiodistal and buccolingual dimensions of all permanent teeth, except third molars, on dental casts of a large sample of 600 individuals from India [28]. They found the canines are the most dimorphic teeth (lower more than upper), and logistic regression analysis allocation accuracy was 68.1% for the maxillary teeth, 73.9% for the mandibular teeth, and 71% for the teeth of both jaws combined. These results are similar to our accuracy rate and point out the lower canines as the most dimorphic teeth.

Research has shown that sex chromosomes influence tooth size. Aneuploidies of the X chromosome affect the production of enamel and the development and pattern of tooth cusps. The excess of X chromosomes in males with Klinefelter syndrome results in thicker enamel and larger teeth, while the lack of the X chromosome in Turner syndrome females results in thinner enamel and consequently smaller teeth [29]. The investigation of crown morphology in a large sample of Turner syndrome and Klinefelter syndrome individuals from the Croatian population showed no difference in expression of the DAR when compared to healthy control individuals [30]. This is one more proof that canine morphology, although it correlates with size, is an independently expressed dental characteristic.

In individuals with a normal karyotype, the sex difference in tooth size is due to thicker dentine, attributable to the promotional effect of the Y chromosome on the mitotic potential of the tooth germ [20,29]. This can also explain why, in males, a longer period is needed for the development of permanent teeth. Greater production of dentine in males contributes to both canine dimensions and morphology.

## 5. Conclusions

Both canine dimensions and morphology proved valuable in sex determination, especially for lower canines. Although the success of the correct classifications is less than in studies where complete dentitions are analyzed, the analysis of canines has the advantage that it can be applicable when dealing with fragmentary human remains and incomplete dentitions. The dental anthropological method of combining odontometry and morphology in an analysis demands experience and practice for achieving accuracy but has multiple advantages: it is simple and achievable using a single tooth—a lower canine—and is non-expensive and non-invasive both for living and archaeological material. Additional value is that it can be applied to children, as lower canine crown formation completes at about 7 years and eruption occurs at about 9 years of age [31]. As Scott and Pilloud [20] note, ancestry should be taken into account when using tooth dimensions to estimate sex. Our research is a contribution to forensic dentistry by providing a discriminant function formula for sex determination in the contemporary Croatian population. Future studies are necessary to test this formula on a different sample of contemporary and archaeological populations and to add new variables that would improve success in sex discrimination.

## Figures and Tables

**Table 1:** Upper and lower canine distal accessory ridge descriptive statistics.

	Sex	n	Minimum	25th Percentile	Median	75th Percentile	Maximum
Upper canine	Males	151	0	2	3	3	5
	Females	140	0	1	3	3	5
Lower canine	Males	154	0	0	1	2	5
	Females	144	0	0	0	1	4

**Table 2:** Sex dimorphism for the upper and lower canine distal accessory ridges as assessed by the Kruskal-Wallis test.

	Upper Canine		Lower Canine	
Test statistic	0.9095		27.9306	
Corrected for ties (Ht)	0.9658		32.5381	
Degrees of Freedom (DF)	1		1	
Significance level (P)	0.325727		**<0.000001**	
Sex	*n*	Average Rank	*n*	Average Rank
Males	151	150.53	154	175.01
Females	140	141.11	144	122.22

**Table 3:** Canine mesiodistal (MD) and buccolingual (BL) crown dimensions; sexual dimorphism tested by Student *t*-test. All *p*-values are statistically significant.

Variable	Males			Females						
	n	Mean	SD	n	Mean	SD	Difference	Diff%	95% CI	*p*
13MD	60	7.89	0.42	60	7.52	0.40	−0.37	−4.63	−0.5129 to −0.2171	**<0.0001**
13BL	60	8.47	0.54	60	8.12	0.51	−0.35	−4.07	−0.5336 to −0.1564	**0.0004**
23MD	60	7.92	0.53	60	7.64	0.42	−0.28	−3.54	−0.4523 to −0.1077	**0.0017**
23BL	60	8.43	0.59	60	8.10	0.54	−0.33	−3.93	−0.5362 to −0.1272	**0.0017**
33MD	60	6.85	0.47	60	6.50	0.41	−0.36	−5.18	−0.5153 to −0.1947	**<0.0001**
33BL	60	7.65	0.58	60	7.41	0.51	−0.23	−3.05	−0.4296 to −0.03708	**0.0202**
43MD	59	6.74	0.47	60	6.39	0.39	−0.35	−5.23	−0.5092 to −0.1954	**<0.0001**
43BL	60	7.69	0.68	60	7.42	0.42	−0.27	−3.47	−0.4713 to −0.06202	**0.0111**

**Table 4:** Discriminant Function Analysis summary with two variables in the final model: lower canine distal accessory ridge (DAR) and lower left canine mesiodistal crown width (33MD).

N = 117	Step 8, N of Vars in Model: 2; Grouping: Sex (2 Groups) Wilks’ Lambda: 0.74554 Approx. F (2.114) = 19.455 *p* < 0.0000					
	Wilks’ (Lambda)	Partial (Lambda)	F-Remove (1.114)	*p*-Value	Toler.	1-Toler. (R-Sqr.)
Lower canine DAR	0.869440	0.857495	18.94535	**0.000029**	0.997851	0.002149
33MD	0.828674	0.899679	12.71189	**0.000532**	0.997851	0.002149

## Data Availability

The original dataset was uploaded as Supplementary Material to this publication.

## Supplementary Materials

The following supporting information can be downloaded at: https://www.mdpi.com/article/10.3390/dj11070175/s1, research dataset “Datasheet Dumancic Canine Crown Sexual Dimorphism”. Table S1: Canine distal accessory ridge scores (ASUDAS DAR) and cervicoincisal (CI), mesiodistal (MD) and buccolingual (BL) dimensions in mm, the FDI tooth notation.
